# A model describing the use of a bronchial blocking device and a sheathed bronchoscope for pulmonary aspiration studies in the Göttingen minipig

**DOI:** 10.1177/0023677213518526

**Published:** 2014-04

**Authors:** E Hulse, F C Reed, M Eddleston, R Etherington, R E Clutton

**Affiliations:** 1 Pharmacology, Toxicology and Therapeutics, Centre for Cardiovascular Science, Queens Medical Research Institute, University of Edinburgh, Edinburgh, UK; 2 Royal (Dick) School of Veterinary Studies, University of Edinburgh, Easter Bush, Roslin, Midlothian, UK; 3 Bristol, UK

**Keywords:** bronchial blocker, aspiration, minipig, bronchoscopy

## Abstract

The administration of test substances into a single lung, or lung lobe, allows the remaining untreated lung to act as an experimental control and effectively halves the number of animals required in a given experiment. It reduces the likelihood of early fatal pulmonary failure when noxious substances are studied which may lessen the need for replacement animals. However, the ease of substance administration and the subsequent analysis of its effects, for example by bronchoalveolar lavage or bronchoscopy, depend critically on the size of the animal model. The advantages of using minipigs; ease of handling, reduced housing requirements, genetic homogeneity, etc. are reduced if their diminutive size makes lung studies difficult. This article describes the use of a bronchial blocking device and a sheathed bronchoscope which enabled sterile endobronchial substance administration in Göttingen minipigs, and allowed pulmonary aspiration studies to be conducted with each animal acting as its own control.

Göttingen minipigs (Ellegaard, Dalmose, Denmark) have been developed as a model for studying the effects of organophosphorus (OP) insecticides in human suicide attempts^1,2^ which account for an annual 300,000 deaths in rural Asia.^3^ The management of patients surviving to hospital admission is often complicated by lung damage caused by the aspiration of the OP itself, its solvent and gastric contents.

Pigs have been used to investigate the effects of gastric contents, toxins, bacterial and viral inoculates, trauma and positive pressure ventilation (PPV) on lung function.^4–12^ They have obvious advantages over smaller rodent models of lung injury including ease of bronchoscopy, segmental bronchoalveolar lavage (BAL) and serial lung biopsies.

In many pulmonary function studies, treatments are applied to both lungs of individual animals and the effects compared with function in untreated controls. The use of bronchial blockers in a selected bronchus ensures treatments are directed to the contralateral lung, permitting the ‘blocked’ lung to act as a control. This effectively halves the number of animals required per study. Bronchial blockers also allow the study of direct (or topical) versus indirect (systemic) effects of treatment on lung structure and function. Similar advantages also result when double lumen endotracheal tubes (DLTs) are used. However, using right-sided DLTs in pigs is likely to obstruct the right accessory bronchus (F; Figure 1) causing right apical lung collapse and increased intrapulmonary shunt. DLTs also require frequent bronchoscopic repositioning over a 48 h period and can themselves cause bronchial damage.

**Figure 1.**
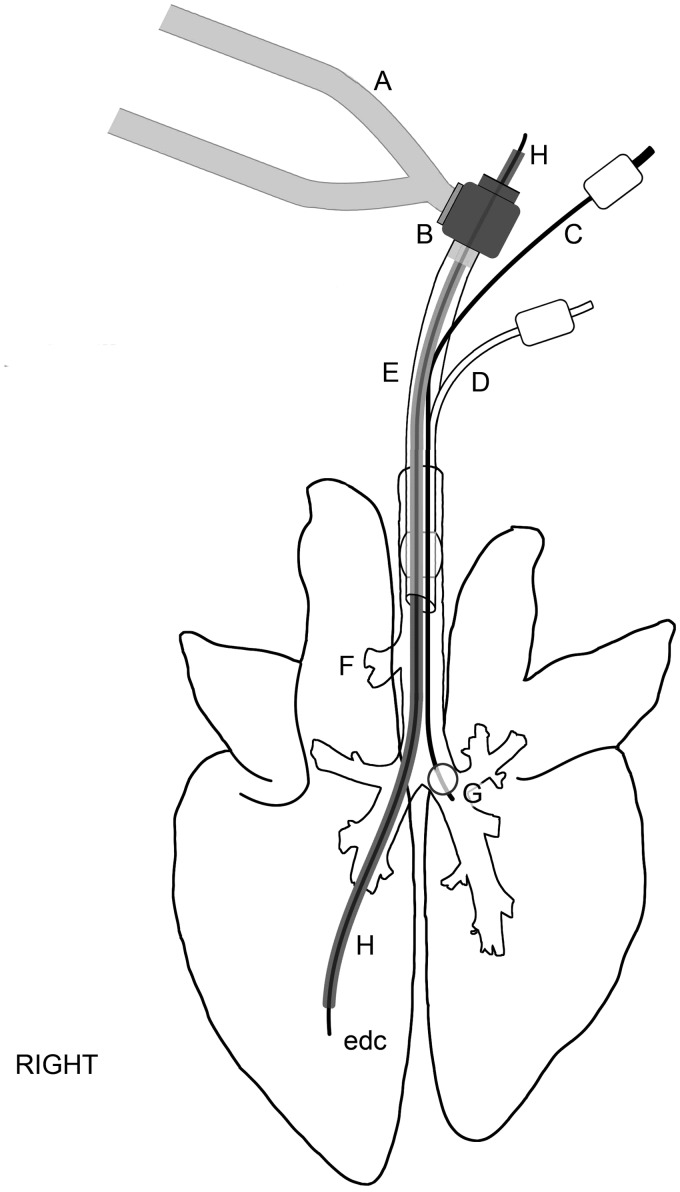
Diagram illustrating the instrumentation used in lung aspiration studies in Göttingen minipigs. The lungs and devices are to approximate scale. A: breathing hoses from Seimens Servo 300 A ventilator; B: bronchoscope dual-axis swivel adapter; C: bronchial blocker inflation line; D: endotracheal tube cuff inflater; E: torque controlled bronchial blocker (TCB; Univent endotracheal tube); F: right accessory bronchus; G: bronchial blocker cuff; H: bronchoscope (BRS-5000); edc: epidural catheter.

The small size of the Göttingen minipig facilitates handling and physical restraint. Yet, bronchoscopy and endobronchial intubation are potentially more difficult than in larger, commercial pig breeds because of the limited airway diameter which is further reduced when orotracheal or tracheostomy tubes are present. The removal of the endotracheal tube (ETT) to facilitate endobronchial examination must be weighed against the benefits of imposing (potentially life-saving) PPV with oxygen between examinations.

Problems with the volume of airway instrumentation and its adverse effects on patency and gas flow are aggravated by measures required to keep the distal airway as sterile as possible. The repeated use of BAL and bronchoscopy increases the risk of iatrogenic infection of the distal airway and necessitates the use of either sterilized bronchoscopes or a bronchial sheath.^13^ Furthermore, the working channel must be of sufficient size for the intended studies, e.g. allowing BAL and/or biopsy.

A torque controlled bronchial blocker TCB Univent tube (Fuji Systems, Tokyo, Japan) was used in conjunction with the bronchoscope (BRS-5000; Vision Sciences, Orangeburg, NY, USA) in 20 anaesthetized Göttingen minipig sows in which the pulmonary effects of instilled gastric juice ± the OP (dimethoate EC40) or solvent (cyclohexanone) were studied over 48 h. This report details the use of these devices and discusses their advantages and disadvantages.

## Materials and methods

### Animals

The study was approved by the Institutional Ethical Review Committee and was licensed under the Animal (Scientific Procedures) Act 1986. Following shipment from Ellegaard Denmark, the 20 Göttingen minipig sows were acclimatized for at least one week in laboratory housing and maintained according to Ellegaard Göttingen minipig^14,15^ and Home Office (UK) guidelines. The mean (range) body mass of the female minipigs was 28.0 (23.5–31.5) kg and they ranged in age from 9–14 months. The animals were housed in groups of 2–6 where ad libitum water was available. Specialized high fibre low-energy feed (Miniporc 801586 SMP; Special Diets Services, Essex, UK) was provided (400–600 g per day). Group sizes were typically four pigs per pen, which were 2 m^2^ and the bedding was wheat straw. Light intensity and duration was 100–200 lux for 12 h. Humidity was held at 50–70% and ambient temperature at 20–22℃. Health checks were conducted daily (at feeding time) and a veterinary inspection was conducted at least once a week. The animals were not fasted and had constant access to straw before being studied.

### Anaesthesia and tracheal intubation

Pre-anaesthetic medication was intramuscular midazolam 0.5 mg/kg combined with ketamine 5 mg/kg. After 12–15 min or when profound sedation was present, the pig was lifted to the surgery table and positioned in sternal recumbency. Anaesthesia was induced using isoflurane from a calibrated vaporizer (vaporizer settings 2.5–4%) delivered in oxygen through a Bain breathing system connected to a Hall pattern mask. Fresh gas flow was 4 L/min in all the animals. The marginal ear vein was cannulated using a 20 or 22 SWG cannula (Vygon, Swindon, UK). When jaw relaxation was adequate, a bandage strip was passed under the maxilla and the head lifted to an angle of approximately 45°. Using a size 4 Miller laryngoscope blade and a Portex stylet, the oropharynx was examined and the soft palate displaced dorsally to reveal the rima glottidis. Topical anaesthesia of the dorsal epiglottic surface and the laryngeal mucosa was then attempted using a 2% lidocaine solution delivered by a laryngo-tracheal mucosal atomization device (LMA MADgic; Teleflex, Hull, UK). The facemask was then repositioned for 45–60 s and the isoflurane oxygen mixture re-administered. Propofol (2 mg/kg), fentanyl (2.5 µg/kg) and rocuronium (1 mg/kg) were injected intravenously, in sequence, over 20, 10 and 2 s, respectively into the auricular venous cannula. The head was lifted as before and the trachea intubated with a size 7.5 or 8.0 mm internal diameter (13 or 13.5 mm outer diameter) TCB Univent tube (Fuji Systems, Tokyo, Japan). Initially, the device was introduced with its concavity directed ventrally but was rotated if the tip failed to advance beyond the level of the vocal folds. Providing no resistance was appreciated, the tube was advanced some 5–10 cm and the bronchial cuff inflated with 10–20 mL air (maximum capacity 50 mL). Correct tube position was confirmed by capnography and the appraisal of chest wall excursions synchronous with those of the preset volume-controlled, pressure-limited mechanical ventilator (Seimens Servo 300 A; Maquet, Sunderland, UK). Thereafter, anaesthesia was maintained using a total intravenous technique, namely propofol (mean 10.6, range (6.8–15.2) mg/kg/h) and fentanyl (mean 5.3, range (2.3–12.2) µg/kg/h).Less anaesthetic was required for OP poisoned pigs secondary to the central nervous system depression. The lungs were ventilated using a mixture of oxygen and medical air (FiO_2_ = 0.5) with a tidal volume of 6–8 mL/kg delivered at 15–25 breaths per minute. Peak inspiratory pressure was limited to <25 cmH_2_O, positive end expiratory pressure (PEEP) was set at 5 cmH_2_O and end-tidal CO_2_ maintained at 5.5 ± 0.8 kPa. The ‘depth’ of anaesthesia was monitored using cranial nerve reflexes, autonomic nervous signs and the bispectral index (BIS; Aspect Medical Systems, Covidien, Dublin, Ireland). Once the animal was anaesthetized, a jugular vein and a carotid artery were surgically exposed and cannulated. Anaesthetic delivery was then switched from the marginal auricular to the jugular vein.

### Endobronchial installation of OP and gastric contents

Before instillation, a bronchoscope (BRS-5000; H; Figure 1) with an external diameter of 5.8 mm incorporating a 2.1 mm working channel was prepared by fitting a sterile sheath (EndoSheath; Vision Sciences) and placing a sterile 16 SWG epidural catheter (Portex Epidural Catheter; Smiths Medical, Kent, UK; edc; Figure 1) approximately three-quarters of the length of the working channel. The catheter connector was fitted to the end of the epidural catheter without a filter. In anticipation of an interrupted oxygen supply and PPV the lungs were ventilated with 100% O_2_ for one minute. Rocuronium 1.0 mg/kg was injected intravenously and allowed 45–90 s to achieve diaphragmatic paralysis. The bronchoscope was then passed through a dual-axis swivel adapter (Portex; B; Figure 1) into the TCB Univent tube until the carina was visible. The bronchial blocker (G; Figure 1) was then advanced approximately 2–4 cm into the left bronchus under bronchoscopic vision using the torque-controlled guide to facilitate positioning. The cuff was then inflated with 2–6 mL of air and the bronchial blocker bung removed to allow gas release from the left lung. The bronchoscope was then advanced through the right main stem bronchus into the right lower lobe until further advance was not possible. At this stage the epidural catheter was pushed down the remainder of the working channel into the right lower lung lobe until further advance became impossible. The syringe containing 0.5 mL/kg of a mixture of gastric juice, saline, OP pesticide and/or solvent was then connected to the epidural catheter and injected.

Further rocuronium was given if bronchospinal reflexes precluded straightforward bronchoscopy. If continuously monitored SpO_2_ fell <80% then the bronchoscope was withdrawn and the lungs ventilated with 100% O_2_ until values had recovered (95–100%).

On completion of injection, bronchoscopy was continued to ensure that the epidural catheter was withdrawn back into the end of the TCB Univent tube, the bronchial blocker cuff was deflated and that the bronchial blocker was fully retracted. The bronchoscope was then removed and PPV resumed with the oxygen/air mixture after closing the bronchial blocker with its bung.

The animals underwent euthanasia on completion of each study while anaesthetized. Pentobarbital (Euthatal; Merial, Harlow, Essex, UK) was injected intravenously (150 mg/kg) and death was confirmed by the absence of vital signs.

### Re-use of the TCB Univent tube

The TCB Univent tubes were re-used for reasons of expense (each tube cost £150–£300). Decontamination of the tubes was followed by sterilization through a 30 min immersion in a 20:1 mixture of water: proprietary sterilant for medical endoscopy and surgical equipment (Medistel; cambridgeshire, UK). The tubes were thoroughly rinsed and flushed with water after immersion and before re-use.

## Results

Sedation was adequate in all cases, with all pigs tolerating mask induction without signs of resentment. Tracheal intubation was achieved without complication in all 20 pigs with the TCB Univent ETT introduced as described. In eight cases the tube had to be rotated through 180° to facilitate passage through the larynx. Laryngospinal reflexes (coughing and ‘bucking’) were encountered in four animals probably because the interval allowed between rocuronium injection and attempted intubation was inadequate (<45 s).

Bronchial blocker placement and installation of the test material into the right bronchus was achieved under bronchoscopy in most cases with ease. However, bronchospinal reflexes were marked even in pigs which hitherto appeared to be adequately anaesthetized on the basis of the absence of jaw tone, palpebral and corneal reflexes. In all cases at least one more injection of rocuronium (0.5–1.0 mg/kg) was required.

Movement of the pigs during repositioning resulted in accidental extubation in three animals. In one case, the trachea was successfully re-intubated without complication; re-intubation was less straightforward in the remaining two as the tubes’ material had softened and rapid repositioning of the animal to facilitate intubation was impeded by experimental instrumentation.

Histopathology scores processed to date show that the mean scores (range 0–18) of 10 pigs were greater in the right lung (direct injury) compared with the left lung (indirect injury) in all but two samples (unpublished data). This supports the belief that the technique described here was effective.

No obvious damage to, or cracking of the tubes appeared as a consequence of repeated (2–4) sterilizations. However, the need to re-inflate the tracheal tube cuffs – sometimes every hour – became increasingly apparent in all the tubes with repeated use, indicating a loss of the cuff material’s integrity under pressure.

Tracheitis was identified in most minipigs at the end of each experiment by bronchoscopy.

## Discussion

The use of the Univent bronchial blocker and the bronchoscope was central to the creation of this model of sterile pulmonary aspiration. Sterile lung injury was achieved using a bronchoscope sheath combined with strict clean animal airway handling and management. The deployment of a bronchial blocker meant that both direct and indirect pulmonary injuries within the same animal could be achieved, and that each animal could be used as its own control.

Tracheal intubation with the TCB Univent tube was straightforward in all cases except on two occasions when re-intubation was attempted. This relative success was pleasantly unexpected and was attributed to the use of the neuromuscular blocking agent (NMBA), rocuronium. NMBAs are not normally used in this laboratory to facilitate endotracheal intubation, and the largest sized ETT that would normally be used in pigs of the size described would have had an internal diameter of 6.0–7.0 mm. That a 13–13.5 mm outer diameter tube was used in 20 animals without inflicting visible tracheal trauma attests to the benefits of using rapid-acting short-duration NMBA for this purpose. However, the use of NMBAs in laboratory animals is rigorously controlled by most licensing authorities because, used improperly, the risk exists that recipients may undergo noxious procedures while conscious but are incapable of motor reaction. This was avoided in the current study by anaesthetizing the animal with isoflurane, de-sensitizing the larynx with lidocaine, ‘deepening’ anaesthesia with propofol and providing further sedation and analgesia with fentanyl before intubation was attempted. Another major problem associated with the use of NMBAs, the paralysis of the respiratory muscles, was circumvented by the imposition of PPV as soon as the airway was secured.

Rocuronium also proved to be useful in preventing coughing in response to bronchial stimulation, which can make endobronchial manipulations more difficult and could cause unwanted injury. Bronchial reflexes could have been suppressed using topical analgesia of the bronchial mucosa with local anaesthetic or by producing deeper levels of general anaesthesia. However, these strategies incur the risk of toxicity and cardiopulmonary depression respectively. The BIS monitor was of little benefit in predicting responses to attempted bronchoscopy: marked responses were observed when BIS values as low as 10 were recorded.

The installation of test material into the airway with subsequent BAL and biopsy could have been achieved using DLTs allowing the ventilation of each lung lobe separately. However, right-sided DLTs can obstruct the right accessory bronchus with subsequent collapse and an increase in shunt fraction. Further, it was felt that the placement and maintenance of a DLT would have been technically more demanding necessitating repeated repositioning with the potential to cause significant bronchial injury over the 48 h period of study. Alternatively, a tracheostomy could have been created which would have allowed the use of larger diameter tubes to facilitate the passage of bronchial blockers. The creation of tracheal stomata to place 8.5–9.0 mm ETTs and accommodate bronchial blockers has been described elsewhere.^16,17^ However, this option was not considered in the current study because lung injury was to be in part quantified by plasma cytokine levels which would have been raised by unnecessary surgery. The decision to use the TCB Univent tube to achieve study objectives was vindicated. The device is an ETT which has a separate lumen within the ETT for a retractable TCB which can be advanced or retracted at any point blindly, or under bronchoscopic guidance.^18^ This option met the study’s objectives although the tubes were large and considerably more expensive than normal ETTs (approximately £150–£300). The former limitation was successfully overcome by the use of rocuronium. An attempt to overcome the latter was made by re-using the devices.

The Univent tube was originally designed for single-use only and its construction precludes straightforward physical cleaning. However, in this study the Univent tubes were cleaned, sterilized and re-used. The main ventilating lumen was easy to clean. The second lumen (containing the bronchial blocker) was more difficult to clean effectively; while Medistel Instrument Disinfectant and water can be forced through the lumen, a brush cannot. In the absence of effective physical cleaning, sterilization depends on the innate sterilization properties of the solution to eradicate organisms and spores. Unfortunately, prolonged repeated exposure to Medistel may degrade the silicone and/or plastic material of the tracheal cuff and its inflation lumen. According to the Medistel data safety information sheet, it is a halogenated tertiary amine with no known adverse effect on non-metallic substances if rinsed after use; however, it can inflame the mucosae if ingested. It is possible that other sterilization methods such as ethylene oxide or chlorine dioxide may be more suitable.

Previous complications with the Univent tube have been documented. Two case reports note that the bronchial blocker cap which prevents air leak when ventilating became detached or fractured during surgery.^19^ This malfunction was not observed during four re-uses of the device in the current study. Similarly, fragmentation of the tube inner wall has been reported^20^ but was not encountered here.

Previous porcine studies describe bronchoscopic placement of bronchial blockers requiring a tracheostomy for larger ETTs to accommodate the bronchoscope.^16,17^ The video bronchoscope (BRS-5000) with sterile disposable sheaths (EndoSheath) in our study allowed rapid and effective instrumentation for pulmonary poisoning and repeated sampling at intervals of 24 and 48 h. The disposable sheaths circumvented the need for long decontamination and potentially ineffective sterilization procedures. However, the sheath itself contains a working channel of 2.1 mm diameter for biopsy forceps and epidural catheters to pass and had an overall diameter of 5.8 mm. This created important parameters because in order to use the bronchoscope effectively and easily the internal diameter of the ETT component had to be >7.5 mm which is why this specific size of Univent TCB was chosen.

The tracheitis observed in most animals could have been caused by regurgitated stomach contents, the toxin installation itself, regular ETT suctioning or possibly a reaction with Medistel traces on the TCB Univent ETT.

In conclusion, the deployment of the Univent bronchial blocker meant that both direct and indirect pulmonary injuries within the same animal could be achieved, and that each animal could be used as its own control. Sterile lung injury was achieved using a bronchoscope sheath combined with strict clean animal airway handling and management. Consideration must be given to the sterilization methods for re-use of the Univent tube to avoid tube degradation and possible tracheitis.
